# Self-Organizing and Scalable Routing Protocol (SOSRP) for Underwater Acoustic Sensor Networks

**DOI:** 10.3390/s19143130

**Published:** 2019-07-16

**Authors:** Sateesh Kumar Hindu, Waheeduddin Hyder, Miguel-Angel Luque-Nieto, Javier Poncela, Pablo Otero

**Affiliations:** 1 Department of Ingeniería de Comunicaciones, University of Malaga, 29010 Malaga, Spain; 2 Institute of Information and Communication Technologies, Mehran University of Engineering and Technology, Jamshoro, Sindh 76062, Pakistan

**Keywords:** multi-hop routing, transmission range, distance, self-organization, energy consumption, end-to-end delay, fault tolerance, isolation recognition

## Abstract

Underwater Acoustic Sensor Networks (UASN) have two important limitations: a very aggressive (marine) environment, and the use of acoustic signals. This means that the techniques for terrestrial wireless sensor networks (WSN) are not applicable. This paper proposes a routing protocol called “Self-Organizing and Scalable Routing Protocol” (SOSRP) which is decentralized and based on tables residing in each node. A combination of the hop value to the collector node and the distance is used as a criterion to create routes leading to the sink node. The expected functions of the protocol include self-organization of the routes, tolerance to failures and detection of isolated nodes. Through the implementation of SOSRP in Matlab and a model of propagation and energy being appropriate for marine environment, performance results are obtained in different scenarios (varying both nodes and transmission range) that include parameters such as end-to-end packet delay, consumption of energy or length of the created routes (with and without failure). The results obtained show a stable, reliable and suitable operation for the deployment and operation of nodes in UASN networks.

## 1. Introduction

Underwater Wireless Sensor Networks (UWSNs) are collections of many autonomous sensor nodes, networked together through wireless links, which perform collaborating tasks to monitor physical or environment parameters such as pressure, temperature, sounds, etc. These networks were initially developed using the concept of terrestrial WSN systems but the fundamental challenges of these two technologies are different. The early implementation of UWSN with radio frequency (RF) and optical links proved that new solutions and approaches are required for underwater environment, which confronts different challenges and limitations in terms of signal propagation, low efficiency of radio wave, transmission range of few meters and scattering in case of optical waves. Therefore, acoustic waves prove to be a promising communication technology, and because of this underwater sensor networks are also referred to as Underwater Acoustic Sensor Networks (UASNs). The use of acoustic communication imposes several constraints, therefore parameters such as carrier frequency, attenuation, noise, fading, propagation delay, and limited bandwidth are important to consider during protocols designing for UASNs. Furthermore, because of water currents and various underwater activities the underwater sensors remain mobile, which makes traditional routing inefficient since the network topology changes over time. Therefore, network topology is also a vital factor in protocol design. Reliability, capacity and energy consumption of network are affected and determined through topology control technique. The reliability of underwater network topology is highly important because of high cost of sensor nodes. Moreover, the propagation environment also has substantial effect on energy consumption which results in node failure because of rapid energy depletion. For this reason, single-point topology should be avoided because a failure in a single node of the network could lead to overall network collapse.

With the advancement in the field of wireless communication and sensor technology, researchers have proposed numerous routing techniques for UASNs [1]. In Reference [2], a new clustering algorithm is proposed using a Low-Energy Adaptive Clustering Hierarchy (LEACH) protocol to address the problem of large clusters and nodes at the edge consuming more energy. In the cluster head (CH) election phase, the position of CH is considered to be the cluster center from the points which were uniformly distributed in the network. For nodes to select the CH, weight factor is introduced which considers the energy consumption between node and CH, and every CH and a base station (BS): It balances the cluster size, reducing the total network energy consumption. Energy Aware and Void Avoiding Routing Protocol (EAVARP) operates in two phases: layering and data collection phase [3]. The sensor nodes are distributed in concentric shells built during the layering phase around the sink. The protocol uses opportunistic directional forward strategy (ODFS) for forwarding the data in a data collection phase to avoid the flooding, cyclic transmissions and voids. The protocol extends the network lifetime through balancing the energy in the network compared to other routing protocols. To address the problem of void nodes and energy-reliability trade off, a Stateless Opportunistic Routing Protocol (SORP) is proposed [4]. The protocol performs a depth-based stateless routing which can avoid the trapped and void areas. It selects the candidate forwarding node through calculating holding time for each node in the forwarding area, using the local information acquired in updating phase from the neighboring nodes. SORP decreases the energy consumption, packet loss and end-to-end delay in all scenarios, sparse or dense. In UWSNs, the efficient data delivering is still a challenge because of limitations of acoustic communication and underwater conditions. To address the packet delivery problem, a hop-based protocol is proposed in Reference [5], known as H^n^ - PERP. The author proposes a centralized model, providing a mechanism for scheduling and data transmission processing. The protocol enhances the energy efficiency and network throughput through power monitoring solutions. In BEEC [6] routing protocol, a circular field is divided into ten sub regions and each region is further divided into eight sectors. The data is collected from the sectors using two mobile sinks, moving in circular patterns where each covers five different sectors in sequence. The protocol increases the performance of network in term of lifetime, energy consumption, throughput and stability. However, the sink nodes follow a fixed circular pattern which leads to packet loss and higher delay because of unawareness of network conditions. Additionally, to collect the data from sensor nodes, Autonomous Underwater Vehicles (AUV) are used to move the sink which requires extra resources to operate the AUVs. A routing algorithm has been proposed through remodeling the Vector-Based Forwarding (VBF) protocol in Reference [7]. It considers the routing pipe radius as a function of node range, number of nodes and dimension of environment. The selection of guiding node is based on the residual energy of the receiving node, increasing or reducing the radius of pipe. The results indicate that the protocol decreases the energy consumption in the network with large number of nodes by changing the routing pipe’s width in proportion to network density. In Reference [8], the proposed protocol routes the data based on its priority. The nodes are deployed in a cube considering the underwater scenario. The algorithm distinguishes the data based on two traffic classes: high priority and low priority. For traffic with high priority, the forwarder node is selected based on the minimum distance to the BS and residual energy present in the target cube. This improves the performance in terms of energy, end-to-end delay and packet loss. In Reference [9], the proposed protocol combines a Depth Based Routing (DBR) protocol with clustering approach to minimize the energy consumption and distribute the load among the nodes in the network. The classification of nodes (normal, CH and dead) in the network is performed based on the assignment of a random number between 0 and 1. In the CH detection phase, if the residual energy of the CH is less than threshold, it will be eliminated as CH and a new CH is formed. The approach has improved the energy efficiency through implementing clustering in depth-based routing. In Reference [10], Energy Efficient DBR (EEDBR) is compared with simple DBR and a hop-by-hop dynamic address based (H2-DAB) protocol. The protocol selects the next node based on lowest depth and highest residual energy from the neighboring nodes. The results show that both path loss and packet delivery ratio are almost same for DBR and EEDBR whereas H2-DAB has a higher end-to-end delay.

The protocol proposed in this work is oriented to the network layer, so it is assumed that a free collision Medium Access Control (MAC) protocol has been previously implemented. There are many techniques applicable for UASNs [11,12,13,14], being TDMA [15,16] one of the most bandwidth efficient. The proposed protocol achieves stability as the network size increases, providing efficient paths to the sink node and fault tolerance through self-organizing the nodes in the network. 

In comparison to Reference [2], SOSRP follows a decentralized mechanism where failure of a single node does not disturb the communications within the network. However, in a clustering approach, the failure of a master node disconnects an entire cluster from the network. SOSRP is a hop-to-hop based communication protocol where nodes relay messages to the sink, whereas in Reference [6] Autonomous Underwater Vehicles (AUV) are required as mobile sinks to collect the data from the deployed nodes. Therefore, extra resources to control the AUVs are needed. In Reference [5], a hop-to-hop based power efficient protocol is proposed. However, the results shown do not present a clear picture of the multi-hop operation on the energy consumed, only the number of hops in the available path.

The paper is structured as follows: Section 2 includes the system model, describing the 3D network model, mathematical model of the propagation, and the energy consumption for undersea environment. Section 3 explains the working methodology of the proposed protocol. Section 4 describes the performance metrics, analysis, and parameters used for evaluation. After that, Section 5 discusses in detail the obtained results in terms of random topology, fault tolerance and scalability. Finally, conclusions are given in Section 6.

## 2. System Model

The system model is a three-dimensional layout including depth due to its impact on important parameters, like energy. A propagation model is implemented considering the underwater conditions and several works proposed by researchers. Moreover, it is worth mentioning that energy consumption is an important parameter to consider in the design of the protocol for any sensor network. Therefore, both models of energy and propagation of acoustic waves have been implemented in Matlab for measuring, including the energy dissipation during the network operation.

### 2.1. Network Model

**Figure 1:**
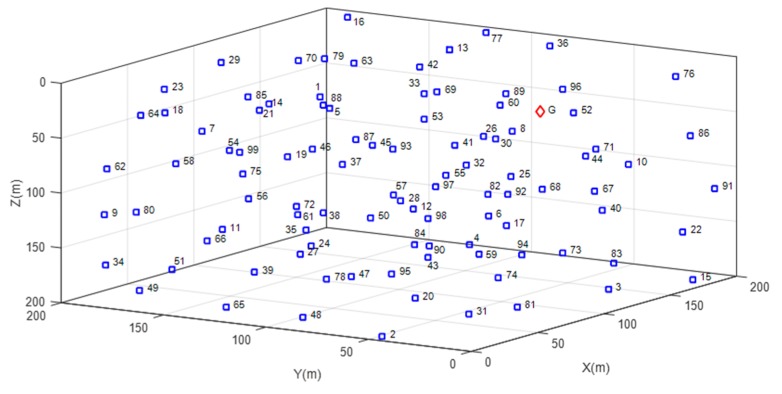
Network Model with 99 sensor nodes (labelled 1–99) and a sink node (labelled G).

The sensor network is 200 × 200 × 200 m cube: The top of cube is considered to be the surface of water and bottom as a seabed. The nodes are deployed one by one in 3D space having random location and depth to address a realistic scenario, including a single sink node on the surface having depth zero. The nodes are randomly placed to assure the flexibility of proposed routing protocol. Once the nodes are placed, they are considered to remain static and do not flow because of marine currents and waves. Each node is placed at a minimum of 40 meters separation from the surrounding nodes. This is done to prevent nodes from sending packets of similar measured events to the sink. The separation between nodes is calculated through Euclidean metric using the well-known equation as follows,

(1) $$d=\sqrt{{\left(x_{2}-x_{1}\right)}^{2}+{\left(y_{2}-y_{1}\right)}^{2}+{\left(z_{2}-z_{1}\right)}^{2}},$$

where ($x_{i},y_{i},z_{i}$) are the location coordinates for the i node. Since the nodes are placed randomly, is assumed that each node uses a power control mechanism to alter and save the transmission power based on the distance between two nodes. Figure 1 presents an example of network model where the nodes are randomly located in 3D space.

### 2.2. Propagation Model

Many characteristics of the underwater environment affect the acoustic communication which makes the propagation channel much more complex compared to the terrestrial communication channel. They include temperature, salinity, multipath fading, path loss, depth and Doppler effect. The acoustic signal propagation, network performance and energy dissipation are highly affected by these factors. According to these considerations, the propagation speed of sound in underwater can be expressed as:

(2) $$\begin{array}{ll} c= & 1448.96+4.591T-5.304*0.01T^{2}+2.374*0.01T^{3}+1.340(S-35)+\\ & 1.63*0.1H+1.675*10^{-7}H-1.025*0.01T\left(S-35\right)-7.139*10^{-13}TH^{3}, \end{array}$$

where *T* is the temperature (in Celsius), *S* the salinity of sea water (in parts per thousands, ppt) and *H* is the depth (m) of sensor nodes.

In UASNs, the signal-to-noise ratio (SNR) is a measure of transmitted signal power to noise power (in dB re 1 µPa, [17]), given by passive sonar equation expressed as [18]:

(3) SNR=SL−TL−NL+DL,

where *SL* is the source level (dB re 1 µPa), *TL* the transmission losses (dB), *NL* the ambient noise (dB re 1 µPa) and *DL* the directivity index (dB). Ambient noise in shallow waters is mainly caused by shipping activity or biological noise, and a suitable level adopted is 70 dB re 1 µPa (50 dB re 1 µPa for deep water). In relation to the source level, is defined as the intensity of sound radiated by source at the distance of 1 meter, expressed in dB re µPa (as [18]) in terms of the Power Intensity ($I_{t}$) (W/m^2^) used:

(4) $$SL=\log10\left(\frac{I_{t}}{0.067*10^{-18}}\right)$$

Hydrophones can be assigned a typical value of 20 dB [18] for SNR, where *DL* is zero when considering omnidirectional modems. Therefore, using (3) *SL* can be written as:

(5) $$SL_{SH}=TL+90$$

(6) $$SL_{DP}=TL+70,$$

where $SL_{SH}$ denotes source level in shallow waters and $SL_{DP}$ source level for deep waters.

Transmission Loss (*TL*) is dependent on the absorption coefficient (α(f), dB/km) and distance. It is the collective depletion in acoustic intensity during wave propagation which significantly affects the underwater communication. Another reason for suffering transmission loss is spreading, with cylindrical shape for a depth lower than 100 meters (shallow waters) or spherical at higher depths (deep waters). In this work, nodes are deployed with random depth from zero to 200 meters so both cylindrical and spherical spreading are considered. In every case, *TL* (dB) can be estimated using (7)–(8) [18]:

(7) $$TL_{CS}=10\log\left(r\right)+\alpha \left(f\right)*r*10^{-3}$$

(8) $$TL_{SS}=20\log\left(r\right)+\alpha \left(f\right)*r*10^{-3},$$

where r is the distance (in meters) between transmitter and receiver, and $TL_{CS}$ and $TL_{SS}$ denotes transmission loss in cylindrical and spherical spreading, respectively. The absorption coefficient α(f) for frequencies ranging from 100 Hz to 10 kHz is expressed through Thorp’s propagation model as:

(9) $$\alpha \left(f\right)=1.094\left(\frac{0.1*f^{2}}{1+f^{2}}+\frac{40*f^{2}}{4100+f^{2}}\right),$$

with frequency (f) measured in kHz.

### 2.3. Energy Consumption Model

The sensor nodes in WSNs are mostly powered through batteries and it is inconvenient to replace it or recharge it when they are depleted. Considering the underwater environment, cost and time required for such operations is high, so energy efficiency is one of the major concerns in designing protocols for UASN. The energy to transmit the data from one node to another over distance is given by Reference [18]:

(10) $$Et=p_{l}*\left(E_{elec}+E_{amp}\right)+P_{t}*\frac{p_{l}}{R},$$

where $p_{l}$ (bits) is the packet length, $E_{elec}$ (J/bit) is the electronics energy consumed, $E_{amp}$ (J/bit) is the amplifier energy dissipation, $P_{t}$ (W) is the power transmitted, and R (bps) is the transmission rate. The $P_{t}$ (W) can be expressed as [18]

(11) $$P_{t}=A*I_{t}=2\pi r*H*I_{t}$$

Similarly, the energy consumed (in J) during reception process is given as [19]:

(12) $$Er=p_{l}*\left(E_{elec}+E_{DA}\right),$$

where $E_{DA}$ (J) is the energy consumed during data aggregation process.

## 3. Proposed Protocol

**Figure 2:**
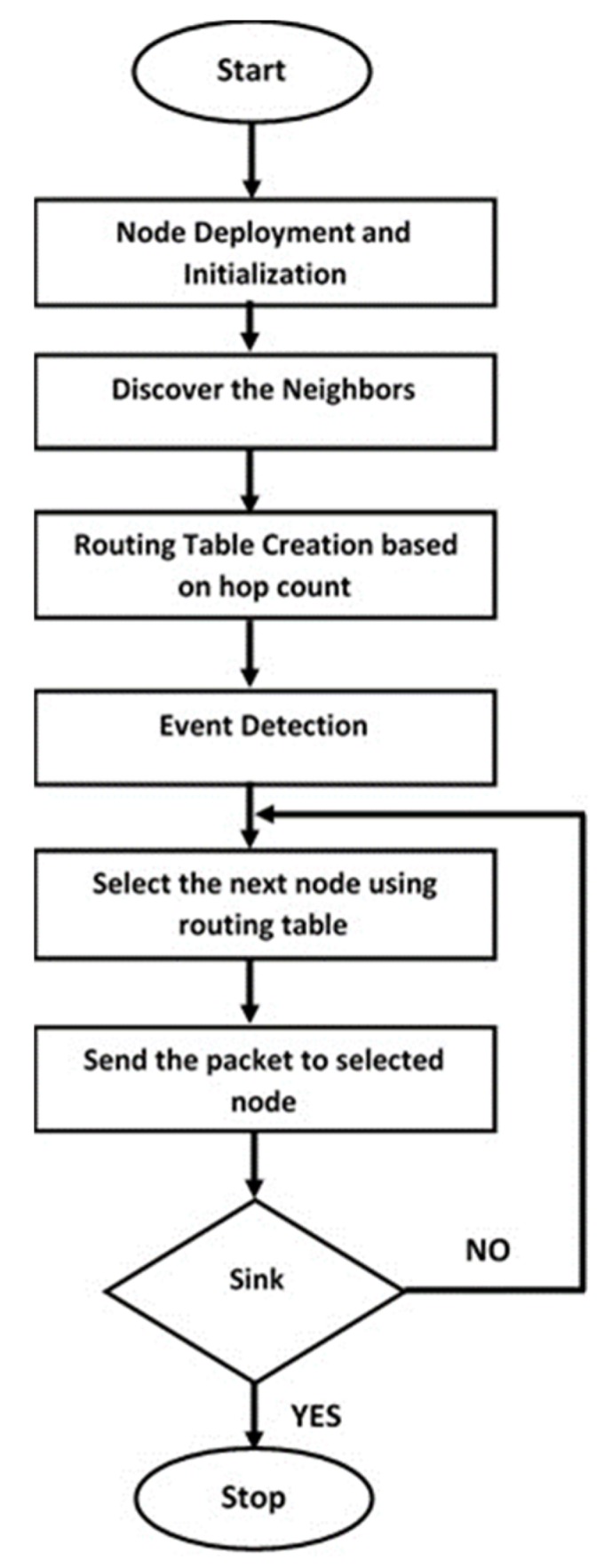
Working methodology of SOSRP.

Considering the challenges, and the harsh ocean environment, a self-organizing protocol is proposed to achieve scalability, robustness and fault tolerant system known as Self Organizing and Scalable Routing Protocol (SOSRP). The SOSRP is designed to conserve the energy and ensure the packet delivery to sink through a power control and hop count-based techniques, including a fault-tolerance algorithm. The protocol enables a node to find the neighboring nodes, forming a connectivity matrix. The packet routing is based on the smallest distance and hop count between the source and sink. Figure 2 depicts the working principle of proposed protocol. The working methodology consists of four phases, chronologically: Network initialization, Neighbor Discovery, Path Selection Criteria and Packet Transmission.

### 3.1. Network Initialization

The nodes are deployed one by one at random depths underwater having random (x,y,z) coordinates, whereas the sink is placed at the surface of sea with zero depth. Initially, after deployment, nodes do not have any prior information about the address and location of the sink node.

In the network initialization phase, the sink node broadcasts a control packet named “HELLO” packet in a defined transmission radius, containing base station ID and hop count which denotes the address and total number of wireless links from node to sink, respectively. After receiving the packet, the node increments the value, stores the hop count if it is not already present or is smaller than the stored hop count and relays the message with the updated value. In the case of the hop count being equal or larger than the current value, the node will discard the message. This process continues until the message reaches every node in the network.

### 3.2. Neighbor Discovery

**Figure 3:**
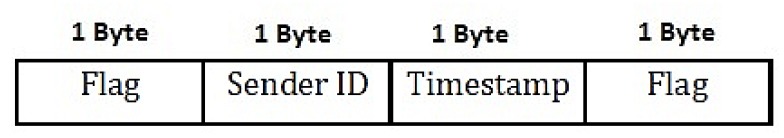
Request Packet Format.

After the initialization phase, a neighbor discovery phase begins where it is considered that each node broadcast a four bytes request message (see Figure 3) in defined transmission range to discover the neighboring nodes. The packet encapsulates sender ID and a timestamp label containing the time when the packet is transmitted.

**Figure 4:**
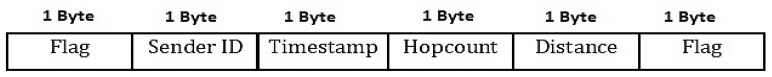
INFO Message Format.

In response, neighboring nodes forwards an “INFO” message of 6 bytes, containing sender/neighbor ID, timestamp, hop count and distance to the sink with a format shown in Figure 4. Upon receiving the packet, the node generates the neighbor table storing neighbor ID, hop count and distance from sink. The Time of Arrival technique is considered for calculating the distance between two nodes and it represents the accumulated hop-to-hop distance from that node to the destination. To conserve the energy, the neighbor discovery phase is only initiated when a change in topology is detected, such as nodes addition or losses.

### 3.3. Path Selection Criteria

The path selection criterion for SOSRP is based on hop count and distance between source and destination. The protocol selects the shortest path between source and sink. On sensing the event, the path formulation begins with the selection of next hop by source node to transmit the data to the sink node.

To diminish the energy consumption during data transmission, the selection criteria of next node is based on smallest hop count and distance from source node to sink node. When source node has data to send, it will look up in its neighbor table to select the next node. The node with the least hop count value will be selected as the next hop. If two neighboring nodes have the same hop count value in its neighbor table, the node with the shortest hop count distance will be selected as the next one. Since, each entry in the table contain an accumulated distance from source to destination as mentioned in Section 3.2, the selected node represents the overall selected path leading to the sink node as an optimal case.

### 3.4. Packet Transmission

In the last phase, the packet is transmitted from source node to the sink using multi-hop communication, where intermediate nodes are selected based on smallest hop count and shortest distance between source and base station.

It is important for a source to select an efficient forwarding node to conserve the energy and minimize the delay. Therefore, on acquiring the data, the source node checks the local routing table for the selection of next hop. The routing table holds neighbor ID, hop count and distance from transmitting node to destination. The forwarding node is selected using path selection criteria where hop count and distance are compared among all the entries stored in the table for the purpose of packet transmission. Upon, selection of forwarding node, source node adds a header containing sink address, selected forwarding node ID, source address/ID, timestamp, hop count and distance. The forwarding node ID is defined for a neighbor to recognize that the packet is meant for it and to avoid other neighboring nodes to transmit it to the sink. As a result, it diminishes the possibility of receiving multiple copies of the same packet. After attaching the header to the data packet, the data is transmitted. Upon reception of packet, the receiving node (selected node) sends an acknowledgement packet to source. This process repeats at each node until the packet reaches the sink node. However, the scope of acknowledgement is limited to hop to hop. If a route failure is detected (i.e., no acknowledgement is received by sending node) the algorithm selects an alternate path to transmit the data based on the routing table information stored in it, repeating the process from the path selection phase.

## 4. Performance Metrics and Analysis

### 4.1. Performance Metrics

The selected metrics to evaluate the performance of SOSRP are energy consumption, end-to-end delay, hop count and number of paths (to the sink). In order to test a stable behavior, two different parameters will be swept: range of transmission and size (number of nodes).

A node dissipates energy in a sensor network while performing the operations necessary for the collection of data required by the application. Such operations include processing, listening to the channel, transmitting, and receiving the data. Energy consumption is the sum of energy dissipation by a node during the process of performing different operations, whereas the accumulation of energy dissipated by each node defines the total energy consumption of network. 

The end-to-end delay refers to the time taken to transmit the data packet from source to sink, irrespective to the number of intermediate nodes. It is the sum of transmission, propagation, queuing, and processing delay in a network.

The hop-count quantity is the measure of number of intermediate nodes between source and destination. In a multi-hop approach, the higher the hop count, the higher will be the energy consumption and end-to-end delay. Therefore, it is essential to keep the hop counter small to lower the delay and energy consumption. Similarly, the hop distance is measured between two neighboring nodes. It is one of the key parameters used to select the next node in the path. Depending on hop distance, the energy is dissipated. Therefore, the smaller the hop distance, the less energy is consumed in delivering the data.

In WSNs, transmission range has significant impact on several parameters of network. It defines the coverage area of a sensor node. If the range of the sensor nodes are large enough to directly reach the sink node (in a hop), they will consume more energy to communicate due to the large distances. However, to converse the energy it is essential to keep the transmission power to a minimum, for which multi-hop (more than a single hop) communication is the best approach. Based on this, transmission power control mechanism is implemented to conserve the energy by calculating the power required to send the packet hop to hop, considering the changing distance between the nodes and receiver’s sensitivity. Different transmission range is tested to evaluate its effects on the performance of network, as can be seen in Section 5.

### 4.2. Analysis

The simulation was tested against different variations in several network parameters and scenarios. Simulation parameters are discussed in Table 1, with some of them taken from References [19,20,21,22]. Considering the different distances between neighboring nodes, the energy and propagation model were simulated to calculate the required power to transmit the packet from one node to another in order to conserve the energy (power control technique).

Two scenarios were considered for simulation: Optimal behavior and Pragmatic behavior. The Optimal behavior presents the flawless path selection and data delivery during the entire simulation time period. Whereas in Pragmatic behavior, a temporary failure is introduced with the probability of 0.2 to test and identify the fault-tolerance mechanism and its effects on the performance metrics selected.

Each scenario was further implemented for both different and same topologies. In different topology, new locations were assigned to nodes with increasing network size, whereas in the same topology, new nodes were added in the existing network keeping intact the previous node locations. In each case, network size and transmission range values were swept to identify (i) the effects of adding new nodes to the network and (ii) the optimal range to use a multi-hop communication. The simulation runtime was 50 rounds; in each round every node sent a single INFO packet containing the sensed data to the sink node.

## 5. Simulation Results

### 5.1. Random Topology

The behavior of the SOSRP was tested with different network sizes (from 50 to 100 nodes). For each network size, new random locations for the nodes were set, and consequently the ad-hoc topology would be different when the number of nodes changed. This was done to observe the effect of newly formed paths. Moreover, the transmission range was also swept (from 70 m to 100 m) to evaluate the effects on the connectivity in the network in terms of three parameters: end-to-end delay, number of hop count in total generated paths, and total energy consumption of the network. The results obtained for the energy consumption are in consonance with other authors [18,19], and the specifications shown in Table 1 can be kept using a range of commercial modems [23,24]. With respect to the packet delay, the time shown in figures needs to be added to the time used by the MAC layer, not considered here in the end-to-end delay. This MAC delay depends of the specific MAC technique employed. 

**Figure 5:**
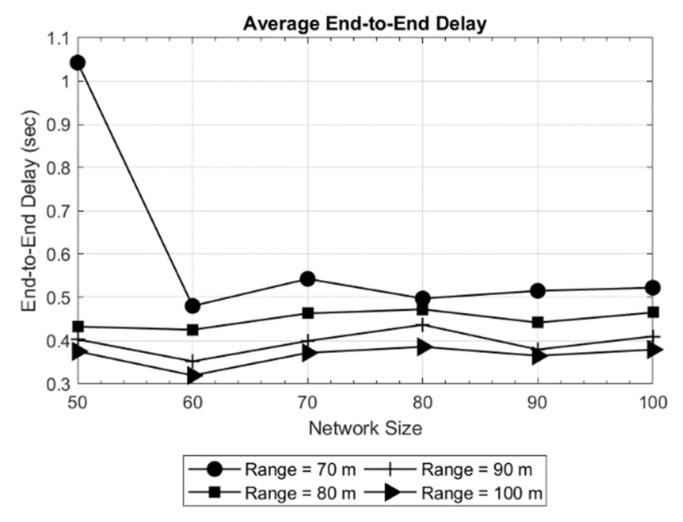
Average delay in network of 200 × 200 × 200 m.

**Figure 6:**
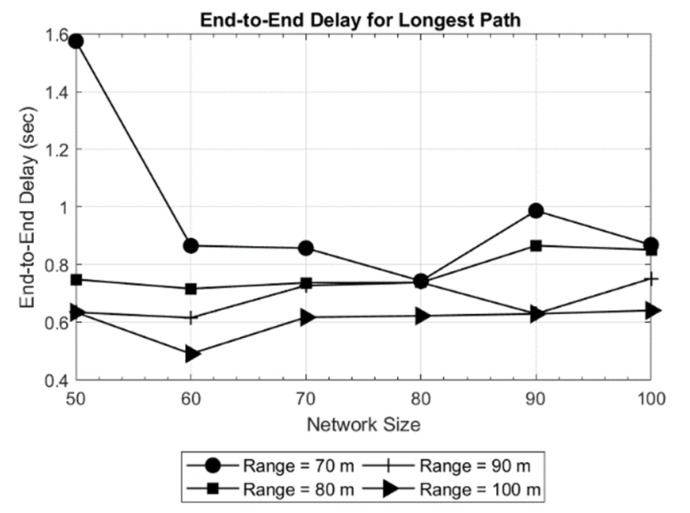
End-to-end delay for longest path in network of 200 × 200 × 200 m.

**Figure 7:**
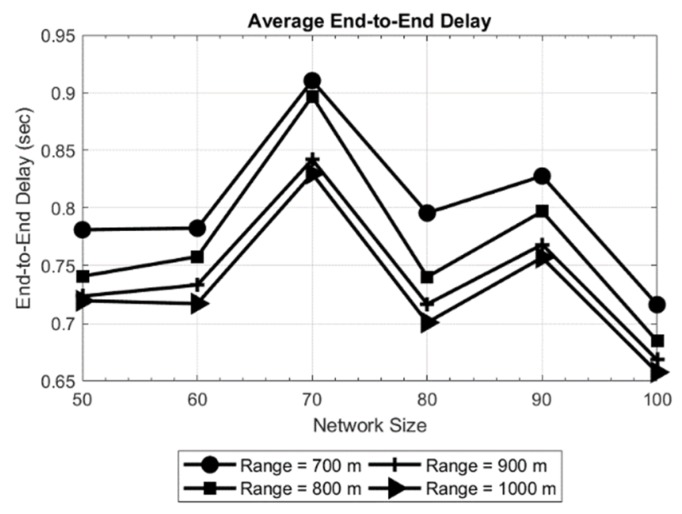
Average delay in network of 2000 × 2000 × 500 m.

**Figure 8:**
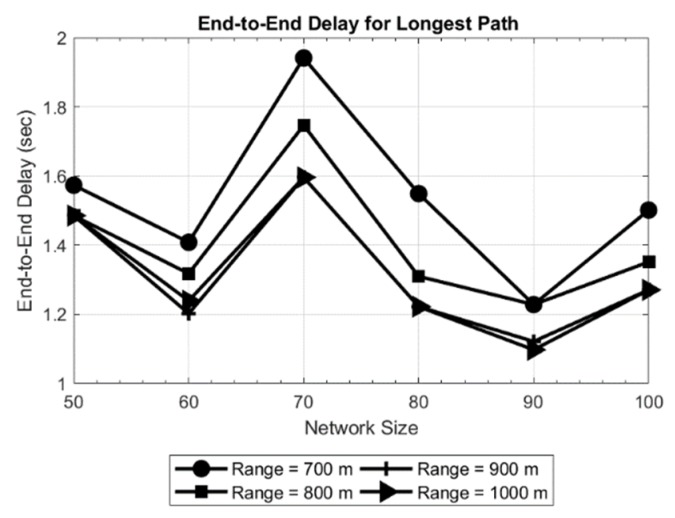
End-to-end delay for longest path in network of 2000 × 2000 × 500 m.

The results obtained are shown in Figure 5 and Figure 6 for a network area of 200 × 200 × 200 m, and in Figure 7 and Figure 8 for a network area of 2000 × 2000 × 500 m, where a stable operation is kept in the network with random topology for each network size. In general, increasing the network size tends to reduce or maintain the end-to-end delay. This means the new routes are converging to the sink in an efficient way. Moreover, a routing delay lower than two seconds for the worst case studied (Figure 8) is not a bad time [18] in such an extensive area of 2000 × 2000 × 500 m.

Another evident result is observed when only the transmission range is increased: the delay decreases. The reason is that the coverage area of the nodes is increased which, in turn decreases the hop count between the source and destination. This effect can be seen in Figure 6, where the delay for the longest path is as high as 1.6 s for a 50-node network and the shortest transmission range (70 meters). Another effect can be witnessed in Figure 8, where in a 70-node network with 700 meters of transmission range, the delay increases with respect to larger networks. This is due to large network area and random placement of node and increasing the number of hops, indicating that SOSRP is adopting the new paths efficiently leading to the sink node. However, a higher transmission range also increases the number of interferences because of spatial overlapping. The MAC implemented must works accordingly to the new situation, avoiding the new possible interferences.

Another parameter to measure the efficiency in multi-hop routing protocols is the hop count in a route (path always ending in the sink node). A low number of hops in every route is desirable to keep a limited end-to-end delay. 

**Figure 9:**
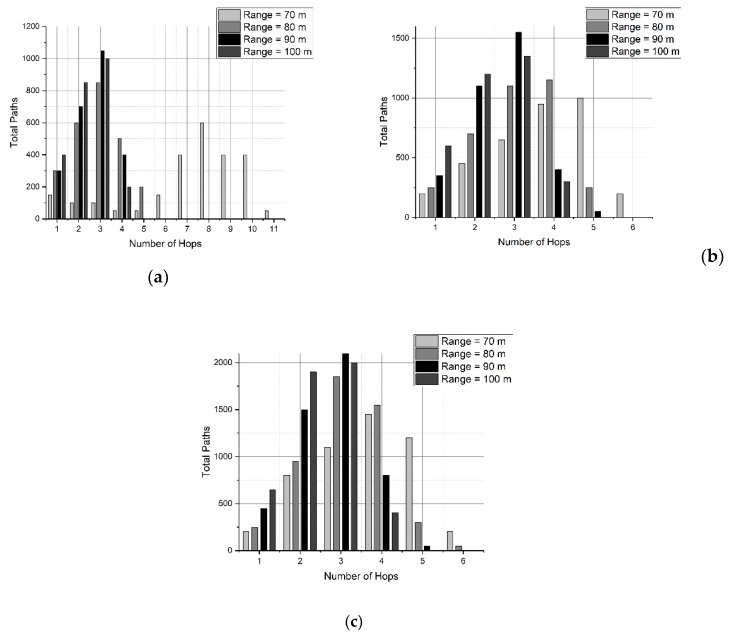
Number of paths for different network sizes (nodes): (**a**) 50, (**b**) 70 and (**c**) 100.

The results of the simulations are shown in Figure 9. In this set of Figures, there is a double interest: to know the number of hops of the routes generated by SOSRP, and to see the influence of the transmission range in this value. Generally, increasing the transmission range leads to the shortest routes (decreasing the hops count). This effect is evident in Figure 9b.

On the other hand, it must be noted that when the network size changes, new random locations are selected for the nodes, changing the topology. For that reason, the result obtained for one network size is not the same case by adding a few nodes. Despite this, the results are consistent with a stable behavior of the SOSRP protocol proposed here.

In order to evaluate the results in terms of energy, we must consider two logical effects. One of them is that as the network size grows, the energy consumption must also increase. The reason is obvious: more nodes are participating in sensing and transmitting data. The second logical effect is related to the transmission range: if it increases, the paths to the sink have fewer hops due to the nodes being not too far from each other. This fact leads us to reduce the energy employed in a small network area such as 200 × 200 × 200 m.

**Figure 10:**
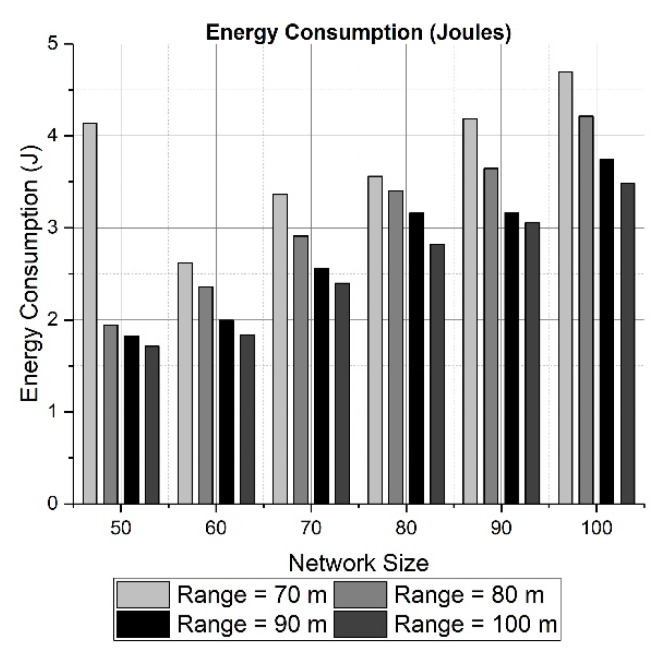
Total energy consumption in 50 Rounds (200 × 200 × 200 m).

**Figure 11:**
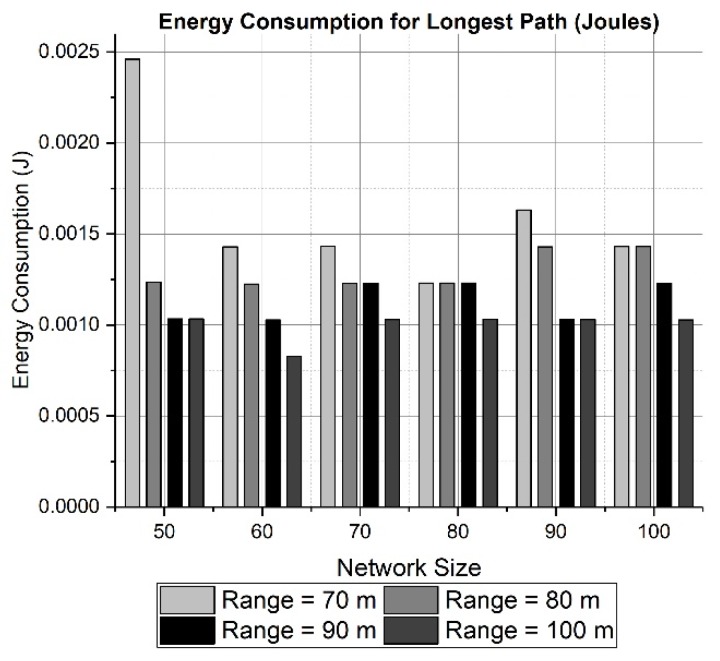
Energy consumption for the longest path (200 × 200 × 200 m).

**Figure 12:**
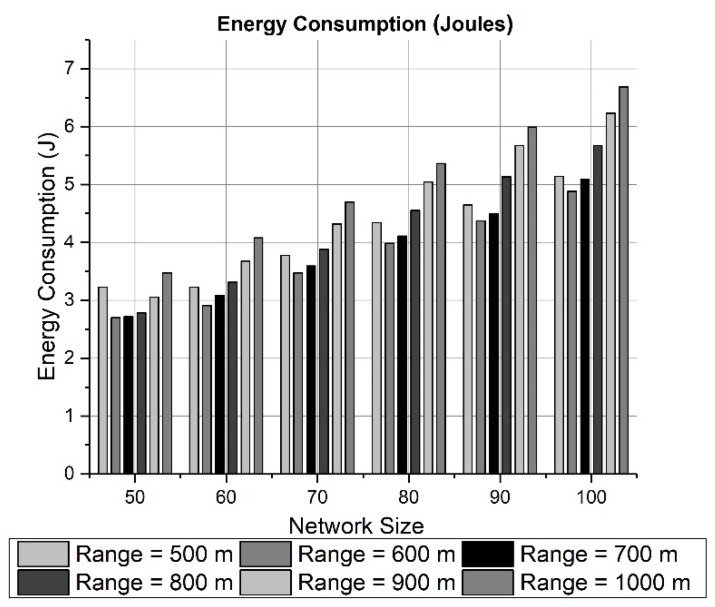
Total energy consumption in 50 Rounds (2000 × 2000 × 500 m).

**Figure 13:**
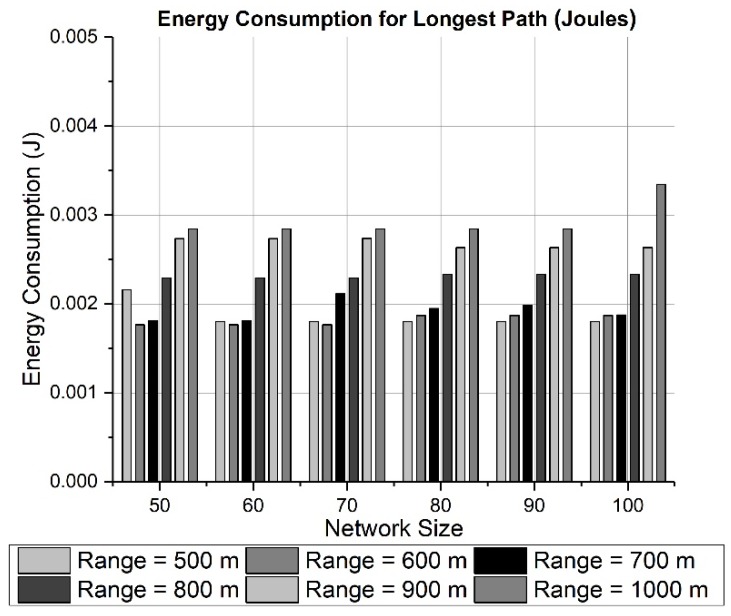
Energy consumption for the longest path (2000 × 2000 × 500 m).

Both effects can be seen in the results presented in Figure 10 and Figure 11 for a network area of 200 × 200 × 200 m. Figure 10 and Figure 12 calculate the total energy consumption for 50 rounds, while Figure 11 and Figure 13 show the energy consumption only for the longest path. The energy consumed for transmitting data through the longest path (Figure 11) has a maximum of 2.5 mJ approximately for a network size of 50 nodes and a transmission range of 70 meters. The effect of choosing a large network area can be witnessed in Figure 12 and Figure 13, where network area is 2000 × 2000 × 500 m. It can be observed in Figure 12 how the energy tends to decrease when the transmission range changes from 500 to 600 meters. However, due to the random placement of nodes in a large area, the distances among nodes are increased. Therefore, the energy consumption also is increased because more power is utilized to communicate with neighboring nodes at farther distance. 

### 5.2. Faulty Tolerance

Because of random deployment of nodes, if a node has *N* number of neighbors. It will have at least *N* number of possible paths leading to the sink. Among the available paths, the one with smallest hop count and shortest distance to sink is considered an optimal path while others are alternate. Alternate path is the best possible route available after optimal path, selected based on the path selection criterion when fault is detected. To further evaluate the fidelity of SOSRP, a fault probability of 0.2 is realized, e.g., in case of 5000 optimal paths found, only 1000 pragmatic paths will be detected. 

**Figure 14:**
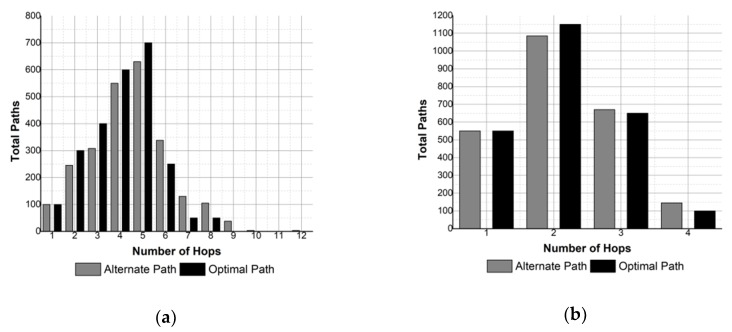
Number of hops for 50 nodes and different transmission ranges: (**a**) 70 m, (**b**) 100 m.

**Figure 15:**
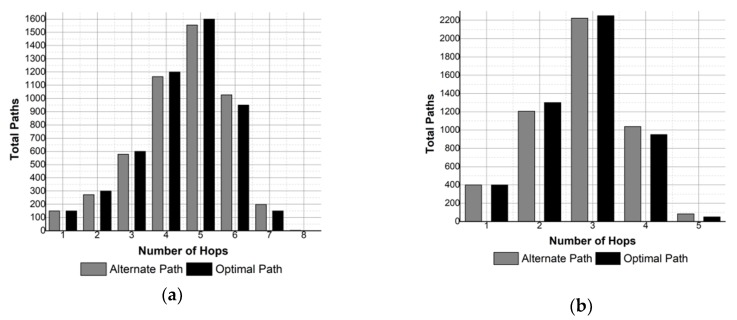
Number of hops for 100 nodes and different transmission ranges: (**a**) 70 m, (**b**) 100 m.

In multi-hop communication, it is desirable to keep the hop count minimum in the path to limit the end-to-end delay and the energy consumption. The results obtained compare the total number of paths generated by SOSRP with number of hops in each path for optimal and pragmatic behavior of protocol. The results are shown in Figure 14 and Figure 15. In Figure 14 the number of hops in the route is shown for a network size of 50 nodes, and 100 nodes in the case of Figure 15.

It is noticeable from the results that SOSRP successfully responds to fault detected in the optimal path by selecting a new route to the sink. However, this increases the number of paths with higher hop count, thus affecting the end-to-end delay and energy consumption of the network.

This effect can be observed in Figure 14a, where 50 nodes are deployed with transmission range of 70 meters. The figure shows the influence of transmission range in pragmatic behavior of network, increasing the hops up to 12 in the alternate path. Similar results can be witnessed in Figure 15a. 

However, increasing the transmission range to 100 meters removes this problem, as shown in Figure 14b and Figure 15b, where the highest number of hops in both optimal and alternate path is four and five respectively, and where pragmatic behavior causes more routes with higher hop count.

**Figure 16:**
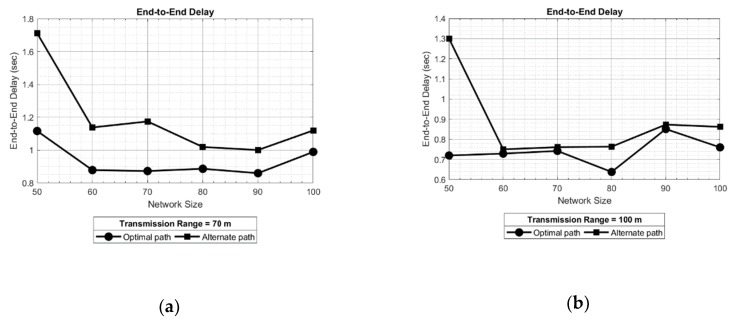
End-to-end delay for optimal and alternate paths for different transmission ranges: (**a**) 70 m, (**b**) 100 m.

The results shown in Figure 16 depict the end-to-end delay of optimal and alternate path, for different network sizes and keeping transmission range 70 and 100 meters. The results show similar behavior of end-to-end delay in alternate path with respect to the optimal path. This effect is evident in Figure 16a. However, it can be also observed that alternate path offers more delay in data transmission as compared to optimal path with a maximum of approximately 1.7 s. Furthermore, increasing the transmission range can mitigate the end-to-end delay because of a reduced number of hops in the newly formed routes. This effect can be observed in Figure 16b for a transmission range of 100 meters, where the obtained delay for alternate and optimal path is approximately 1.3 and 0.7 s respectively, which is much less than the delay observed in Figure 16a.

**Figure 17:**
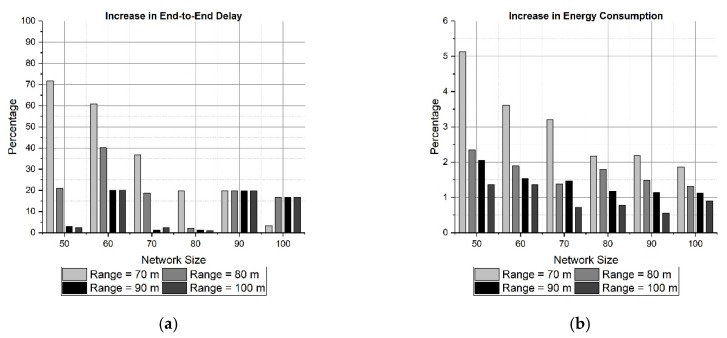
Increase (%) in maximum end-to-end delay (**a**) and in energy consumption (**b**).

In order to examine the performance of SOSRP, the percentage of increase for end-to-end delay and energy consumption is calculated. It is performed by measuring the two parameters for optimal and alternate path. The results obtained are shown in Figure 17.

Figure 17 shows the percentage of increase in maximum end-to-end delay and total energy consumption, obtained in pragmatic behavior of protocol. The influence of increasing the transmission range and network size is obvious in both cases (a) and (b), reducing the percentage of end-to-end delay and energy consumption. This low increment indicates a proper operation of SOSRP.

### 5.3. Network Scalability

In a real UASN, when it is needed to increase the number of nodes, it would be very expensive and illogical to collect out of the water those in service and make a new deployment again one by one until the total number of sensors is completed. Instead of this, a more realistic task would be to perform a new deployment of only the new nodes needed, maintaining the topology of the previous network.

**Figure 18:**
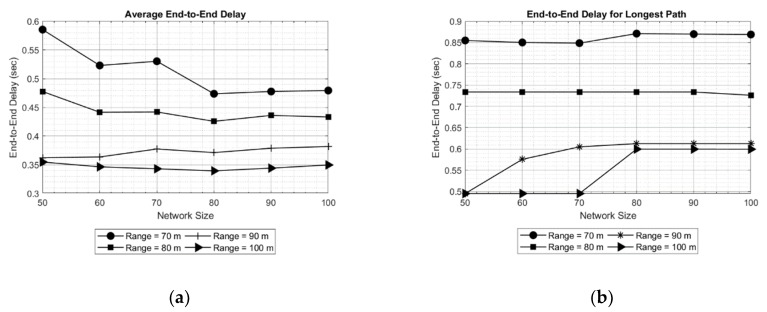
Average end-to-end delay (**a**) and end-to-end delay for longest path (**b**).

**Figure 19:**
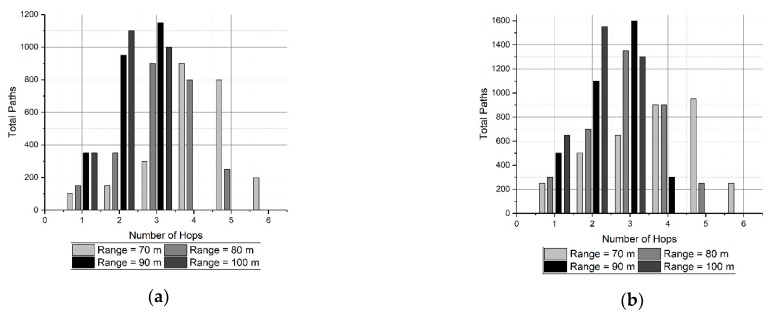

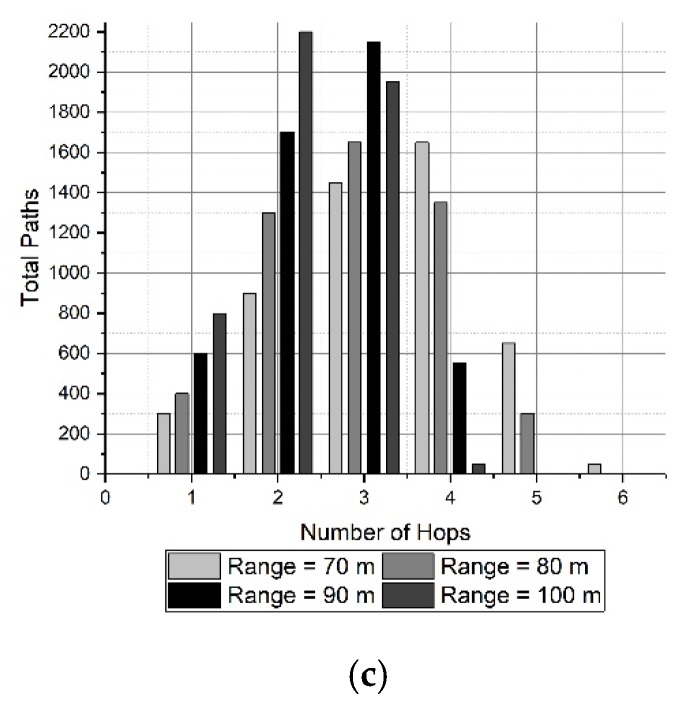
Number of hops in each path vs transmission range for three network sizes: (**a**) 50, (**b**) 70, (**c**) 100.

Considering the above discussion, SOSRP was tested for network scalability by deploying the 50 nodes at first and simulating them for different ranges (70 to 100 meters). Ten new nodes were randomly added to the network in each simulation, keeping the previous location of deployed nodes. The process was repeated until the network size reached 100 nodes. This approach is more realistic and allows each newly deployed node to connect with the network using the local information from neighbor nodes. Moreover, the number of operations decreased in the network (e.g., calculating new routes) by keeping the routes stable with minimum changes when few nodes were added to the existing network. It is essential for the network to perform the necessary functions irrespective of variation in the number of nodes. Figure 18 shows the outcomes of average (a) and longest path (b) end-to-end delay. As previously discussed, it is obvious from the figures that with increasing transmission range the end-to-end delay decreases because of lower number of hops in the selected route. However, with changing network size there is a slight variation in delay. This stability in delay is observed by preserving the routes stable (previous topology) and newly deployed nodes within the transmission range of other nodes, keeping the maximum number of hops the same as before. This effect can be further seen in Figure 19.

The results show the number of hops in all paths generated for different network sizes (50, 70, and 100). It is quite evident from the results that by adding new nodes in the network, the total number of paths is increased because of the addition of ten nodes in each run. However, the maximum number of hops (max: hops = 6) in any path are the same for different network sizes, as shown in Figure 19. This proves that performance metrics of SOSRP remain stable irrespective of increasing network size. 

**Figure 20:**
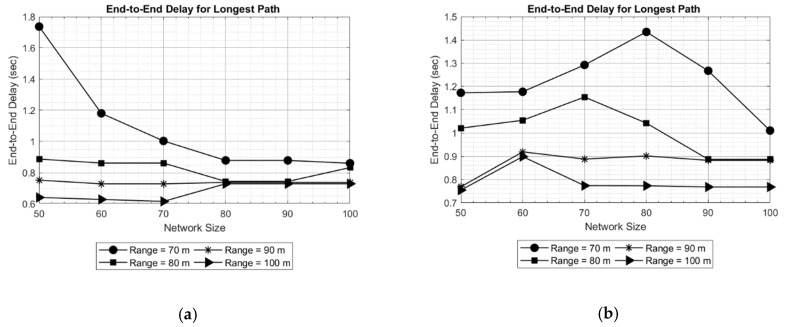
End-to-end delay for longest path fortwo particular simulations: Test 1 (**a**) and Test 2 (**b**).

In order to validate the observed results, multiple simulations were performed. The results of two simulations (Test 1 and Test 2) are shown in Figure 20 for end-to-end delay obtained in longest path. Comparing the obtained results, it can be seen in Figure 20a that with increasing network size, the delay for longest path decreases for a transmission range of 70 meters. An opposite effect can be observed in Figure 20b, where delay is increasing up to a network size of 80 nodes, keeping the transmission range 70 meters. This is because of random placement of new nodes, increasing the number of hops in the route selected. However, the maximum longest path delay obtained in Test 2 (approximately 1.45 s) is still less than maximum delay in Test 1. The stability is obtained in longest path delay by increasing the transmission range. The effect is noticeable in results for different transmission ranges (80, 90 and 100).

## 6. Conclusions

With advancements in the field of wireless communication and sensor technology, new techniques and protocols are proposed for UASNs. These kinds of networks have become popular among researchers because of applications such as disaster prevention, ocean exploration and resource discovery. Different centralized and distributed network routing approaches have been proposed by researchers to make the communication efficient in underwater.

In this paper, using the concept of decentralized network a Self-Organizing and Scalable Routing Protocol (SOSRP) is proposed where each node forms a local connectivity based on the information acquired from the neighboring nodes and performing the data transmission. The protocol utilizes a multi-hop communication technique to transmit the sensed data to the sink node. Each node formulates the routing table using control packets broadcasted in initialization and neighbor discovery phase, and path selection is based on the information of hop count and distance to base station (sink node) from the transmitting node. The Matlab platform was used to simulate the protocol along with both proper energy and propagation models for acoustic communication to contemplate the undersea conditions. The performance of the protocol was measured against end-to-end delay, energy consumption and number of hops in the path by varying network size and transmission range for optimal and pragmatic behavior of SOSRP. Through different simulations, it was found that an optimal transmission range and network size can improve the performance of the protocol by decreasing the number of hops in the generated path. The results show that SOSRP provides stable operation, scalability, fault tolerance, and isolation detection for UASNs.

## Figures and Tables

**Table 1:** Simulation Parameters.

Notation	Parameters	Value
-	Simulation Rounds	50
-	Network Area (m)	200 × 200 × 200
-	Network Size	50, 60, 70, 80, 90, 100
-	Transmission Range (m)	70, 80, 90, 100
SNR	Signal Noise Ratio (dB)	20
NL_SH_	Noise level (Shallow Waters, dB re 1 µPa)	70
NL_DP_	Noise level (Deep Sea, dB re 1 µPa)	50
f	Frequency (kHz)	20
BW	Bandwidth (kHz)	4
E_elec_	Electronics Energy (nJ)	50
E_amp_	Amplifier Energy (nJ)	0.0013
E_idle_	Idle State Energy (nJ)	30
T	Temperature (Celsius)	20
S	Salinity (ppt)	34
p_dl_	Data Packet Length (bytes)	240
p_H_	HELLO Packet (bytes)	4
p_INFO_	INFO Packet (bytes)	6
p_REQ_	Request Packet (bytes)	4
R	Transmission rate (kbps)	26.6
